# Therapeutic relationship elements and therapy session outcomes: Protocol for a longitudinal study of the patient’s perspective.

**DOI:** 10.12688/openreseurope.16466.1

**Published:** 2023-09-01

**Authors:** Alberto Stefana, Paolo Fusar-Poli, Eduard Vieta, Eric A. Youngstrom

**Affiliations:** 1 Department of Brain and Behavioral Sciences, University of Pavia, Pavia, 27100, Italy; 2 Department of Psychosis Studies, Institute of Psychiatry, Psychology & Neuroscience, King's College London, London, England, SE5 8AF, UK; 3 OASIS Service, South London and Maudsley NHS Foundation Trust, London, SE5 8AZ, UK; 4 Bipolar and Depressive Disorders Unit, Hospital Clinic, IDIBAPS, CIBERSAM, University of Barcelona, Barcelona, Catalonia, 08028, Spain; 5 Helping Give Away Psychological Science, Chapel Hill, North Carolina, 27599, USA; 6 Department of Psychology and Neuroscience, The University of North Carolina at Chapel Hill, Chapel Hill, North Carolina, 27599, USA

**Keywords:** Therapeutic relationship, psychotherapy, adult patients, session outcome, patient’s perspective, scale validation

## Abstract

**Background:** There is a growing recognition of the key role of the therapeutic relationship in the outcomes of psychotherapy. However, current understanding of its specific components, their interplay and related patient-therapist dynamics is limited.

**Objective:** (a) To validate two self-report measures to assess subjective affective reactions of patients toward their psychotherapists during specific therapy sessions, and (b) to explore the relationships and dynamics among four elements of the therapeutic relationship: patient reactions toward the therapist, working alliance, alliance ruptures and repairs, and the real relationship.

**Methods:** This study uses a nonrandomized, two-time point longitudinal design. The target population is adult patients currently engaged in individual psychotherapy for heterogeneous mental conditions. Participants are recruited through two online recruitment platforms: Research for Me and ResearchMatch. Data collection involves administering two surveys through the Qualtrics online survey platform. The baseline survey assesses information about the most recent therapy session and the preceding week, while the follow-up survey collects data on the subsequent therapy session and the days leading up to it.

**Discussion:** This research offers three main contributions: (a) it furthers evidence-based assessment in psychotherapy by creating and validating two novel, succinct self-report tools; (b) it enhances theoretical understanding within therapeutic relationship research by exploring the significant impact of patients' perceptions of relationship elements on session outcomes variability; and (c) it will identify therapeutic relationship elements that can either enhance or hinder the overall relationship quality and session outcomes.

**Ethics and dissemination:** The study protocol was approved by the Institutional Review Board of the University of North Carolina at Chapel Hill. The results will be published in indexed peer-reviewed journals and presented at relevant psychology and psychiatry conferences.

## Background and rationale

The therapeutic relationship between patients and therapists plays a crucial role in the unfolding of the psychotherapy process and contributes significantly to the treatment outcomes, regardless of the therapeutic techniques and models used ( Gelso, 2018; Norcross & Lambert, 2018). Growing empirical evidence emphasizes the therapeutic relationship, pointing to the theoretical and clinical importance of broadening the investigation to underexplored or unexplored aspects of this crucial relationship ( Bhatia & Gelso, 2018). Such a valuable shift from a broad analysis of the general therapeutic relationship to a more focused investigation of its specific components, as well as their interplay and their relationships with patient, therapist, and treatment variables, will improve our understanding of the psychotherapeutic relationship.

Among the patient’s characteristics, the mediating role of personality traits in shaping the therapeutic relationship appears particularly intriguing, although it is not yet fully understood ( Dennhag *et al.*, 2017; Fletcher & Delgadillo, 2022). Furthermore, although evidence emphasizes the influence of both patient and therapist contributions to the therapeutic relationship ( Norcross & Lambert, 2019; Wampold & Imel, 2015), the existing literature focuses mainly on therapist variables, often neglecting the patient’s unique perspective, especially with respect to their affective and cognitive responses during sessions ( Norcross & Lambert, 2018). This is noteworthy because among the emotions experienced by patients in the “here and now” of the therapeutic session, those directed at the therapist are particularly significant and valuable for the fulfillment and facilitation of the therapeutic work in question ( Høglend, 2014; Subic-Wrana *et al.*, 2016).

Interestingly, although various measures have been developed to assess emotions ( Norcross & Lambert, 2019; Peluso & Freund, 2018), only a few of them explicitly incorporate specific attention to affective processes within the dyadic therapeutic relationship ( Stefana *et al.*, 2023a). Existing self-report measures that capture therapeutic relationship constructs such as alliance ( Wampold & Flückiger, 2023) only indirectly address affective content. Therefore, it would be valuable to have scales optimized to focus on affect in the context of ongoing therapy.

### Study objectives

This study has two primary objectives:

a. To validate the *in-Session Patient Affective Reactions Questionnaire* (SPARQ) and the *Rift In Session Questionnaire* (RISQ) devised by Stefana *et al.* (2023). These self-report measures assess the subjective affective reactions of patients toward their psychotherapists during specific therapy sessions. The study aims to establish the reliability and validity of these measures through a rigorous psychometric evaluation.
b. To explore the relationships and dynamics among four elements of the therapeutic relationship: patient reactions toward the therapist, working alliance, alliance ruptures and repairs, and the real relationship. The study aims to investigate how these elements interrelate and how they collectively influence the outcomes of the therapy session, from the patient’s perspective. Furthermore, the study aims to examine possible moderating effects of patient, therapist, and process characteristics on the strength of the therapeutic relationship and the outcome of the session.

## Methods

### Study design

This study employed a nonrandomized, two-time point longitudinal design. The study started in March 2023 and is ongoing. The initial development of the scales in Stefana *et al.* (2023) used a large initial item pool and a large, single administration sample to generate adequate sample size to use exploratory and confirmatory factor analysis and item response theory to guide item selection. A limitation of that work was that the SPARQ and RISQ items were originally embedded in a larger item pool. It is possible that the items would perform differently in the shorter, extracted format that would be more useful in clinical practice and therapy research. A second limitation is that each participant responded only once, so it was not possible to estimate score stability or sensitivity to change. The approach used in the present study addresses both limitations of the prior work: (a) it collects cross-sectional data for the validation of the SPARQ and the RISQ in the extracted, brief item sets, and (b) gathers longitudinal data to examine how specific elements of the therapeutic relationship are related to each other and to the outcome of the therapy session, from the perspective of patients. Furthermore, it allows us to test the moderating role of personality characteristics of the patients.

### Participants

The target population for this study includes adults (aged 18 years and above) seeking treatment for heterogeneous mental conditions who are currently under individual psychotherapy. Participants must be fluent in English and capable of providing informed consent.

Participants are recruited through two online recruitment platforms: Research for Me and ResearchMatch. Research for Me has approximately 12,000 registered volunteers in North Carolina, while ResearchMatch ( Harris *et al.*, 2012) provides access to more than 155,000 volunteers across the United States. These platforms connect researchers with individuals interested in contributing to scientific advancements in various fields. The use of the extensive reach and user-friendly interfaces of Research for Me and ResearchMatch improves participant recruitment for this study, ensuring a representative sample from diverse backgrounds and geographic locations.

### Sample size estimation

The study includes two main goals, and the necessary sample size varies accordingly.

The first goal is validation of the psychometrics of the SPARQ and RISQ in their brief, extracted item sets. Based on the number of items in the measures to be validated ( *k* = 8 for the SPARQ and *k* = 4 for the RISQ), as well as the expected moderate to strong factor loadings (as found in the embedded format study; Stefana *et al.*, 2023), a sample size of 700 would be considered a “very good” sample size according to established guidelines for scale development ( Comrey & Lee, 2013). This sample size ensures excellent power to capture the true factor structure ( MacCallum *et al.*, 1999) and allows for more than acceptable comparability of patterns ( Guadagnoli & Velicer, 1988). The sample size considered for this study is deemed feasible based on the previous psychotherapy-related study of our team that successfully used the same two online recruitment platforms.

For the second goal, evaluating prospective associations among scores rated over the course of treatment, and the potential moderating effects of personality, even using conservative estimates that only 70% of cases would complete the follow-up ratings, power would be 80% to detect effects as small as *f ^2^* = .02 (alpha = .01, two-tailed, *N* = 500, and including up to 9 predictors or covariates) ( Faul *et al.*, 2009), corresponding to a small effect size using Cohen’s ( Cohen, 2013) benchmarks.

### Data collection

Data collection involves administering two surveys through Qualtrics, a secure online survey platform hosted on the University of North Carolina at Chapel Hill server. The baseline survey assessed information about the most recent therapy session and the preceding week, while the follow-up survey collected data on the subsequent therapy session and the days leading up to it (see Table 1).

**Table 1.** Measurement tools and timeline.

Domains and measures	Temporal reference	Baseline at session X	Follow-up at session X+1
*Demographic and therapy domain*			
Sociodemographic, clinical, and treatment data form	treatment epoch	✓	
*Personality domain*			
Big Five Inventory–2-Extra-Short form	trait	✓	
The Level of Personality Functioning Scale–Brief Form 2.0	trait	✓	
*Mental health state domain*			
International Positive and Negative Affect Schedule-Short Form	state–session	✓	
Patient Health Questionnaire–9	state–session	✓	
Generalized Anxiety Disorder–7	state–session	✓	
Single-item global measures of symptom severity, psychosocial functioning, and quality of life	state–session	✓	✓
*Therapeutic relationship domain*			
Real Relationship Inventory–Client form	current relation	✓	
Working Alliance Inventory–Short Revised	state–session	✓	✓
Patient Session Questionnaire	state–session	✓	✓
in-Session Patient Affective Reactions Questionnaire	state–session	✓	✓
Rift In-Session Questionnaire	state–session	✓	✓
*Session outcome domain*			
Session Evaluation Scale	outcome–session	✓	✓

### Measures

This study employs a comprehensive battery of measures to collect a wide array of data related to the therapeutic process and patient factors. The demographic and therapy domain of the patient encompasses the sociodemographic details of the patient and the psychotherapy details. The personality domain refers to measures of personality trait characteristics. The mental health state domain refers to the traditional measures of symptoms that currently affect the participants day to day life. The therapeutic relationship domain will evaluate specific elements of the in-session relationship between the patient and the therapist.

### Data form domain

***Sociodemographic, Clinical, and Treatment Data Form.*** This form captures self-reported information on the patient’s age, biological sex, gender identity, educational background, ethnicity, any existing psychiatric diagnoses, and the duration and frequency of the current psychotherapeutic intervention. It also records the modality of session attendance (i.e., in-person, video call, or telephone call), the setting of therapy (for example, private practice, private healthcare institution, university counseling center), and the biological sex of the therapist.

***Personality domain.*** The *Big Five Inventory–2-Extra-Short form* (BFI-2-XS) ( Soto & John, 2017) is a concise 15-item self-report instrument that captures personality at the level of the Big Five domains. Each domain is represented by three items, and each item is rated on a 5-point Likert scale. The five domains include extraversion, agreeableness, conscientiousness, negative emotionality (also referred to as neuroticism), and open-mindedness. The average alpha reliability for the BFI-2-XS domain scales fell between .61 and .63, with the total range extending from .51 to .72. Test-retest reliabilities for these scales averaged .70 in the university sample (ranging from .60 to .80) and .76 in the college sample (with a range of .71 to .80).

The *Level of Personality Functioning Scale-Brief Form 2.0* (LPFS-BF 2.0) ( Weekers *et al.,* 2019) is a 12-item self-report questionnaire used to assess the severity of personality pathology. It measures impairment in self-functioning and interpersonal functioning based on levels of personality functioning according to the DSM-5. Cronbach’s alpha for the LPFS-BF 2.0 was .82 for the total scale, .79 for the self-functioning scale, and .71 for the interpersonal functioning scale.

***Mental health state domain.*** The *International Positive and Negative Affect Schedule–Short Form* (I-PANAS-SF) ( Thompson, 2007) is a 10-item self-report measure that captures the frequency of positive and negative affects experienced over the past week. It provides an understanding of the emotional states of the patients. The I-PANAS-SF positive affects and negative affects subscales showed Cronbach’s alphas of, respectively, .78 and .76.

The *Patient Health Questionnaire–9* (PHQ-9) ( Kroenke *et al.*, 2001) is a depression screening tool that captures the severity of depressive symptoms over the past 7 days. It can be used to evaluate symptoms according to the DSM-5 criteria. The internal reliability of the PHQ-9 in the primary care sample was Cronbach’s alpha of .89.

The *Generalized Anxiety Disorder–7* (GAD-7) ( Spitzer *et al*., 2006) is a 7-item self-report measure of anxiety symptoms, with a focus on generalized anxiety disorder. It captures the severity and frequency of anxiety symptoms over the past 7 days. The internal consistency of the GAD-7 was Cronbach’s alpha of .92.

The three *Single-item global measures of symptom severity, psychosocial functioning, and quality of life* (SI) originally developed by Zimmerman *et al.* ( Zimmerman *et al.*, 2006) were used in this study with adaptations. Specifically, “symptoms of depression” was replaced with “symptoms for which you are in psychotherapeutic treatment.” The symptom severity scale uses a 5-point Likert scale ranging from 0 ( *None*) to 4 ( *Severe*). The psychosocial functioning scale similarly uses a 5-point scale from 0 ( *Not at all*) to 4 ( *Extremely*). The quality of life scale ranges from 0 ( *Very good, my life could hardly be better*) to 4 ( *Very bad, my life could hardly be worse*).

***Therapeutic relationship domain.*** The *Real Relationship Inventory–Client* (RRI-C) *form* ( Kelley *et al.*, 2010) is a 24-item self-report instrument that assesses the strength of the “real relationship,” referring to the genuineness of interactions and the perception of each other’s reality in the therapeutic relationship. It includes two subscales: Genuineness and Realism, each capturing distinct aspects of authenticity and perceptual accuracy in the therapy relationship. Internal consistency estimates Cronbach’s alpha for the RRI-C were .95 for the total score, .91 for the Genuineness subscale, and .90 for Realism subscale.

The *Working Alliance Inventory–Short Revised* (WAI-SR) ( Hatcher & Gillaspy, 2006) is a succinct self-report instrument consisting of 12 items to assess the quality of the therapeutic alliance. The measure is divided into three subscales, each with four items, which focus on agreement on therapy tasks, goals, and the development of an emotional bond between the patient and the therapist. With a 6-point Likert scale, it quantifies the degree of agreement, with higher scores indicating a more robust therapeutic alliance. The alpha reliabilities for all WAI-SR subscales spanned a range of .85 to .90, while the total score alpha reliabilities were recorded at .91 and .92.

Part B of the *Patient Session Questionnaire* (PSQ) ( Samstag *et al.*, 1998) is a four-item self-report instrument measuring therapeutic alliance ruptures and their resolution during the most recent session. It includes an initial item that investigates the presence (“Yes” or “No”) of any tension, conflict, or misunderstanding in the therapist-client relationship during the session. If such an event occurred, it is followed by three items that gauge the peak intensity of the tension, the degree to which the problem was addressed in that session, and the patient’s perception of the solution of the problem by the end of the session.

The *in-Session Patient Affective Reactions Questionnaire* (SPARQ) ( Stefana *et al.*, 2023) is a patient-reported tool that comprises 8 items that explore the patterns of thought, feeling, and behavior activated and experienced by the patients toward their therapist during a session. It consists of two scales: Positive Affect and Negative Affect. The positive affect scale exhibited a Cronbach’s alpha coefficient of .86 and an average inter-item correlation of .61. On the other hand, the negative affect scale demonstrated an alpha of .74 and an average inter-item correlation of .41.

The *Rift In-Session Questionnaire* (RISQ) ( Stefana *et al.*, 2023) is a 4-item self-report questionnaire designed to measure the patient’s risk of experiencing ruptures in the therapeutic relationship. The RISQ measures feelings of belittlement, rejection, disparagement, and attack. The RISQ demonstrated good internal consistency with a Cronbach’s alpha coefficient of .75 and an average inter-item correlation of .43.

***Session outcome domain.*** The *Session Evaluation Scale* (SES) ( Lent *et al.*, 2006), part of the Helping Skills Measure ( Hill & Kellems, 2002), evaluates the quality of the therapy session from the patient’s perspective. It consists of five items, four of which are rated on a 5-point Likert scale, and the fifth item gauges the effectiveness of the session. The 5-item SES showed a Cronbach’s alpha coefficients ranging from .88 to .89 ( Lent *et al.*, 2006).

### Efforts to minimize potential sources of bias

In this study, comprehensive measures were implemented to address potential biases. First, to ensure a representative sample, participants were recruited through ResearchMatch, a national online platform with a wide and diverse volunteer base across the United States. This strategy mitigates selection bias and improves the generalizability of the study. Second, we adopted a gender-sensitive approach, emphasizing non-binary gender as a pivotal variable in both analyses and interpretation of the results, ensuring more robust scientific quality and clinical relevance. Third, our longitudinal design, capturing data at two distinct time points, serves dual purposes: validating the SPARQ and RISQ, and examining score stability over time, thus reducing temporal biases. Fourth, our comprehensive set of measures encompasses various therapeutic and patient factors, ranging from sociodemographics to personality traits, reducing measurement bias by offering a holistic view of the therapeutic context. All data are collected through Qualtrics, a secure online platform that preserves participant confidentiality and minimizes response biases. Lastly, meticulous sample size estimations were made, accounting for attrition and ensuring statistical power, thereby preventing biases from under-powered analyses or inadequate representation.

### Data analysis

Regarding the first objective, the analysis plan for validating the two scales includes several statistical techniques to evaluate the psychometric properties. In the initial stage, will be used to evaluate the suitability of the proposed factor models on the complete sample of participants. These will use the same analytic methods used in the original scale development, but with the extracted, short-form subset of items (versus the prior work, where these items were embedded in the larger original item set). Specifically, a two-factor model representing positive affect and negative affect will be tested for the SPARQ, while a single-factor model will be applied to the RISQ ( Stefana *et al.*, 2023). Item response theory (IRT) models will be used to analyze the options characteristics of each scale and obtain detailed information at the item level (including item difficulty, discrimination, and category functioning). In addition to these specific analyzes, several statistical procedures will be conducted to evaluate the psychometric properties of the scales. Internal consistency will be assessed using Cronbach’s alpha (for comparability with what is most often reported for other scales), McDonald’s omega total coefficients (which is more appropriate for measures that include more than one factor), and average inter-item *r* (which avoids confounding internal consistency and scale length, which is a well-known limitation of Cronbach’s alpha; Streiner *et al.*, 2015).

Criterion validity will be examined by calculating the correlations between the scales and various external variables, including sociodemographic, clinical, and treatment variables. Validated measures of traits, mental state characteristics, specific elements of the therapeutic relationship, and session outcomes will also be included in the analysis. Fisher’s z transformation will be used to average pooled correlation matrices, providing descriptive summaries for associations with sets of variables.

Regarding the second aim, correlations will quantify the pre-post stability of the repeated measures. The PROCESS macros will test for mediation of pre-post symptom severity, psychosocial functioning, and quality of life by ratings of the client-therapist relationship, affect, and working alliance, as well as the extent to which these might show moderated mediation due to demographic or personality variables ( Hayes, 2022). Given the number of analyses, alpha will be .01, two-tailed.

## Study status

At the time of submission of this protocol article, the baseline assessment is complete and the follow-up data collection is almost complete.

## Ethical considerations

### Research ethics approval

This study was approved by the Institutional Review Board (IRB) of the University of North Carolina at Chapel Hill (IRB number: 23-0216; approval dated: 06 March 2023). The study was designed and conducted in accordance with ethical guidelines for human subject research.

### Consent or assent

Participants in the study must provide their informed consent in electronic form prior to participation. A consent statement is presented at the start of the initial online survey, allowing participants to choose between “I consent to participation in this study” or “I do not consent to participation in this study.” Those who selected the latter option are automatically redirected to a closing page. The consent statement ensured that the participants are adequately informed of the purpose of the study, the procedures, potential risks and benefits, the confidentiality of the data and their rights as study participants, including the right to withdraw from the study at any time without any consequences.

### Access to data

Access to the data is restricted to the core research team. External requests for access to anonymized data will be considered after completion of the study and subsequent primary publications. The requests must comply with data protection regulations and the objectives and methods of the proposed research must be scientifically and ethically sound.

## Dissemination policy

The findings from this study will be published as preprints and then disseminated through peer-reviewed publications and conference presentations. Study measures, scoring instructions, copies of the syntax for the analyses run, and copies of the presentations and preprints will be stored in a repository on the Open Science Framework (OSF.io). Wikiversity pages, specifically crafted to provide technical resources for both clinicians and researchers, will include links to this repository. The results of the study may also be shared with relevant mental health organizations and used to inform future research and potential improvements in therapeutic relationships in psychotherapy. We aim for a comprehensive and inclusive dissemination strategy to reach academics, clinicians, and the public.

## Discussion

The present research will make three main contributions. (a) Advancing evidence-based assessment in psychotherapy. The findings of this research will contribute to the validation of two innovative, concise self-report instruments that assess the unique facets of the therapeutic alliance from the point of view of the patient. The project advances prior work by focusing specifically on the shortened versions that will be most useful for clinicians, patients, and clinical research. These tools will be made accessible at no cost for clinical and research applications. (b) Theoretical contribution to therapeutic relationship research. This study will enrich the existing body of knowledge on therapeutic relationships, allowing a deeper understanding of how patients’ perceptions of elements of the therapy relationship contribute significantly to the variability in session outcomes. Our findings will also offer insight on the influence of patient personality traits and current mental status on the quality and strength of the therapeutic relationship and the quality of the session outcome. (c) Clinical contribution. Understanding the interplay between specific components of the therapeutic relationship (particularly, real relationship, alliance, and emotional responses toward the therapist), patient characteristics and state, and session outcome will allow clinicians to be aware of the elements of the therapeutic relationship that can either improve or undermine the quality of both the overall therapy relationship and the session outcome.

## Ethics and consent statement

This study was approved by the Institutional Review Board (IRB) of the University of North Carolina at Chapel Hill. Participants in the study provided their informed consent in electronic form prior to participation.

## Data Availability

No data are associated with this study.
