# Supplementary material for: Therapeutic relationship elements and therapy session outcomes: Protocol for a longitudinal study of the patient’s perspective
Source: Open Res Eur. 2024 Jun 4;3:133. Originally published 2023 Sep 1. [Version 2] doi: 10.12688/openreseurope.16466.2 (PMC11310652; doi:10.12688/openreseurope.16466.2)
Supplement: Peer review history

# Reviewer report: Reviewer response for version 2

**DOI:** 10.21956/openreseurope.19211.r41214

**Published:** 2024-08-08

**Authors:** Jacopo Lisoni

**Affiliations:** 1 ASST Spedali Civili of Brescia, Brescia, Italy

The Authors clearly replied to all the questions raised, modifying the manuscript accordingly. Now the protocol has definitely improved as a whole.

Is the study design appropriate for the research question?

Yes

Is the rationale for, and objectives of, the study clearly described?

Yes

Are sufficient details of the methods provided to allow replication by others?

Yes

Are the datasets clearly presented in a useable and accessible format?

Yes

Reviewer Expertise:

My expertise is on personality disorders and on brain stimulation interventions in psychiatry

I confirm that I have read this submission and believe that I have an appropriate level of expertise to confirm that it is of an acceptable scientific standard.

# Reviewer report: Reviewer response for version 1

**DOI:** 10.21956/openreseurope.17775.r39619

**Published:** 2024-05-06

**Authors:** Camellia Hancheva

**Affiliations:** 1 Sofia University, Sofia, Bulgaria

The article "Therapeutic relationship elements and therapy session outcomes: Protocol for a longitudinal study of the patient's perspective" focusses on the importance of the therapeutic relationship from the patient perspective and how it is connected to the outcomes of psychotherapy. The planned study aims to validate two self-report measures which assess patients' affective reactions towards their therapists during specific therapy sessions. The study also seeks to investigate the relationships and dynamics among four elements of the therapeutic relationship: patient reactions towards the therapist, working alliance, alliance ruptures and repairs, and the real relationship. The research utilizes a nonrandomized, two-time point longitudinal design with adult patients engaged in individual psychotherapy for various mental conditions. Data is collected through online surveys administered via the Qualtrics platform, focusing on information from recent and upcoming therapy sessions. The study aims to contribute to evidence-based assessment in psychotherapy, enhance theoretical understanding of therapeutic relationships, and identify elements that impact relationship quality and session outcomes.

 The objectives of the study are clearly described and the design is adequately chosen and elaborated to provide plausible answers to the research questions. Methodology is clear and sufficient details for replication of the study are provided.

 One minor stylistic remark:

 Even though "real relationship" is a phrase from a name of one of the instruments described in the methodology there might be some ambiguity and misunderstanding how "real relationship" is assessed by a subjective one person perspective. I would recommend a bit more extensive explanation /definition of what is implied by "real relationship" or substitution of the phrase with "genuineness and realism in the relationship"

 This article and the planned study would provide valuable insights into the crucial role of the therapeutic relationship, and subjective patient’s affective responses in psychotherapy outcomes. The validation of two self-report measures to assess patients' affective reactions towards their therapists is a significant contribution to evidence-based assessment in the field. Introduction of a sound psychometric instruments in the study of psychotherapeutic process would be beneficial for both clinical practice and research. By exploring the relationships among various elements of the therapeutic relationship, the study enhances theoretical understanding and sheds light on how patients' perceptions impact session outcomes. The focus on identifying elements that can either enhance or hinder relationship quality and session outcomes is particularly insightful. Overall, this research is a valuable addition to the literature on therapeutic relationships in psychotherapy and has the potential to inform clinical practice and enhance individualized treatment decisions.

Is the study design appropriate for the research question?

Yes

Is the rationale for, and objectives of, the study clearly described?

Yes

Are sufficient details of the methods provided to allow replication by others?

Yes

Are the datasets clearly presented in a useable and accessible format?

Not applicable

Reviewer Expertise:

My research is focused on therapeutic process and mediators of therapeutic outcomes as well as assessment of effectiveness of manualized therapeutic interventions. I also work in cultural adaptation of psychometric instruments.

I confirm that I have read this submission and believe that I have an appropriate level of expertise to confirm that it is of an acceptable scientific standard.

# Reviewer report: Reviewer response for version 1

**DOI:** 10.21956/openreseurope.17775.r35244

**Published:** 2023-09-29

**Authors:** Jacopo Lisoni

**Affiliations:** 1 ASST Spedali Civili of Brescia, Brescia, Italy

The study aims to further evidence-based assessment in psychotherapy by validating two self-report tools, to enhance theoretical understanding within therapeutic relationship research and to identify therapeutic relationship elements that can influence the relationship quality and session outcomes.

 Methodologically, the study is very rigorous. The writing of the article is standardized. The aims are clearly described.

 I have only some points that in my opinion deserve further clarification.

1. In the study purposes, the authors declared that study aims to explore the relationships and dynamics among four elements of the therapeutic relationship: patient reactions toward the therapist, working alliance, alliance ruptures and repairs, and the real relationship. I suggest to add a brief discussion of these elements of the therapeutic relationship to increase readability in the Introduction.
2. According to the first goal (validation of the psychometrics of the SPARQ and RISQ), no detailed information on power analysis for Sample size estimation were provided. Please fill this gap.
3. In the Methods, the authors reported that “The target population for this study includes adults (aged 18 years and above) seeking treatment for heterogeneous mental conditions who are currently under individual psychotherapy”.This is a broad definition. It would be desirable for the authors to define more clearly what they mean for “heterogeneous mental conditions”. beyond depressive and anxious disorders, are people with psychosis, PTSD, ODC and others also included? this is an important matters as psychological approaches differ for these disorders. 
4. When describing “measure”, an overview of the *Session outcome domain *is lacking when, instead, it is presented for the other domains
5. In**** *the Sociodemographic and therapy domain **, *** it is not clear if the authors collected additional data on the type/approach of psychotherapy (and not only data on the duration and frequency of the current psychotherapeutic interventions). In other words, do the Authors have considered the fact that the type of psychotherapeutic approach could be another factor that could influence the therapeutic relationship? I think that it’s certainly true that in all psychotherapy approaches the relationship is fundamental, but the type of emotional response that is elicited in the patient is certainly different depending on the approach used. Is there any evidence that different therapeutic approaches could influence the therapeutic relationship?
6. According to the third aim of the study (to identify therapeutic relationship elements that can influence the relationship quality and session outcomes), do the Authors have consider to perform ad hoc analyses on the influence exerted by a specific mental condition (depression, anxiety disorder, personality disorder etc.)?

Is the study design appropriate for the research question?

Yes

Is the rationale for, and objectives of, the study clearly described?

Yes

Are sufficient details of the methods provided to allow replication by others?

Yes

Are the datasets clearly presented in a useable and accessible format?

Yes

Reviewer Expertise:

My expertise is on personality disorders and on brain stimulation interventions in psychiatry

I confirm that I have read this submission and believe that I have an appropriate level of expertise to confirm that it is of an acceptable scientific standard, however I have significant reservations, as outlined above.

# Response

**Published:** 2024-05-09

**Authors:** Alberto Stefana

**Affiliations:** Department of Brain and Behavioral Sciences, University of Pavia, Pavia, Italia, Italy

Thank you for your positive feedback and your comments. We have included for you below a point-by-point response.  

**Reviewer: **In the study purposes, the authors declared that study aims to explore the relationships and dynamics among four elements of the therapeutic relationship: patient reactions toward the therapist, working alliance, alliance ruptures and repairs, and the real relationship. I suggest to add a brief discussion of these elements of the therapeutic relationship to increase readability in the Introduction.

**Authors: **A definition of these constructs has been added in the Therapeutic relationship domain section.  

**Reviewer: **According to the first goal (validation of the psychometrics of the SPARQ and RISQ), no detailed information on power analysis for Sample size estimation were provided. Please fill this gap.

**Authors: **A sample size estimation has been added.  

**Reviewer: **In the Methods, the authors reported that “The target population for this study includes adults (aged 18 years and above) seeking treatment for heterogeneous mental conditions who are currently under individual psychotherapy”.This is a broad definition. It would be desirable for the authors to define more clearly what they mean for “heterogeneous mental conditions”. beyond depressive and anxious disorders, are people with psychosis, PTSD, ODC and others also included? this is an important matters as psychological approaches differ for these disorders. 

**Authors: **It has been explicated that there were no restrictions on the type of mental disorder. **Reviewer: **When describing “measure”, an overview of the *Session outcome domain *is lacking when, instead, it is presented for the other domains

**Authors: **It has been added.  

**Reviewer: **In****** *the Sociodemographic and therapy domain* ***, *** it is not clear if the authors collected additional data on the type/approach of psychotherapy (and not only data on the duration and frequency of the current psychotherapeutic interventions). In other words, do the Authors have considered the fact that the type of psychotherapeutic approach could be another factor that could influence the therapeutic relationship? I think that it’s certainly true that in all psychotherapy approaches the relationship is fundamental, but the type of emotional response that is elicited in the patient is certainly different depending on the approach used. Is there any evidence that different therapeutic approaches could influence the therapeutic relationship?

**Authors: **It has been specified that no information about the specific types of psychotherapy that participants were undergoing was gathered.

**Reviewer: **According to the third aim of the study (to identify therapeutic relationship elements that can influence the relationship quality and session outcomes), do the Authors have consider to perform ad hoc analyses on the influence exerted by a specific mental condition (depression, anxiety disorder, personality disorder etc.)?

**Authors: **This will depend on the availability and adequacy of the final dataset. Specifically, we will need to ensure that there are sufficient subjects within each diagnostic category to conduct the analyses effectively.
